# Evaluation of the Add-On Effect of Chinese Patent Medicine for Patients with Stable or Unstable Angina: A Systematic Review and Meta-Analysis

**DOI:** 10.1155/2013/673193

**Published:** 2013-12-12

**Authors:** Chen Mao, Vincent C. H. Chung, Jin-Qiu Yuan, Yuan-Yuan Yu, Zu-Yao Yang, Xin-Yin Wu, Jin-Ling Tang

**Affiliations:** ^1^Division of Epidemiology, JC School of Public Health and Primary Care, The Chinese University of Hong Kong, Prince of Wales Hospital, 4/F School of Public Health Building, Shatin, New Territories, Hong Kong; ^2^Shenzhen Municipal Key Laboratory for Health Risk Analysis, Shenzhen Research Institute, The Chinese University of Hong Kong, 10 Yuexing Erdao, Nanshan District, Shenzhen, Guangdong 518000, China; ^3^The Hong Kong Branch of the Chinese Cochrane Centre, Faculty of Medicine, The Chinese University of Hong Kong, Shatin, New Territories, Hong Kong

## Abstract

Chinese herbal medicine (CHM) has been widely used as an adjunct to western medicine in treating angina in China. We carried out this systematic review to evaluate the effectiveness of CHM on top of western medicine for angina. This meta-analysis included 46 randomized control trials with 4212 patients. For trials that included stable angina patients, the CHM group had significant lower incidence of total heart events (relative risk (RR) = 0.50, 95% confidence interval (CI) 0.33–0.78), myocardial infarction (RR = 0.32, 95% CI 0.14–0.72), heart failure (RR = 0.37, 95% CI 0.15–0.91), and angina (RR = 0.46, 95% CI 0.30–0.71) than that of control group. For trials that included unstable angina patients, CHM led to significantly lower occurrence of total heart events (RR = 0.46, 95% CI 0.32–0.66), myocardial infarction (RR = 0.37, 95% CI 0.26–0.54), and angina (RR = 0.36, 95%CI 0.26–0.51). Likewise, for trials that included stable or unstable angina patients, the rates of myocardial infarction (RR = 0.34, 95% CI 0.17–0.68) and angina (RR = 0.46, 95% CI 0.30–0.70) in CHM group were significantly lower than that in control group. In conclusion, CHM is very likely to be able to improve the survival of angina patients who are already receiving western medicine.

## 1. Introduction

Angina is pain or constricting discomfort that typically occurs in the front of the chest and is brought on by physical exertion or emotional stress [1]. It is the main symptomatic manifestation of myocardial ischemia, caused by an imbalance between myocardial blood supply and oxygen demand [1, 2]. The prevalence of angina in the population appears to increase in the past decades [3]. A meta-analysis of data from 31 countries indicated the population weighted prevalence was 6.7% in woman and 5.7% in man [4]. Angina is a common initial presentation of coronary disease [5], and it may exert a major impact on quality of life, ability to work, and costs to society [6, 7].

Angina is clinically classified into stable angina (SA) and unstable angina (UA), and treatment strategy is different between them. SA is a chronic medical condition and the aim of management for it is to abolish or minimize symptoms, improve quality of life, and decrease long-term morbidity and mortality [1] while UA is an acute coronary syndrome, which should be treated as an emergency [8]. The current antiangina medical management includes pharmacological strategies, revascularization strategies, and lifestyle interventions. Chinese herbal medicine (CHM) is also widely used for treating angina in China [9].

Because angina is a life-threatening event and very effective western medicine treatments are available, CHM is usually used in addition to baseline treatment with western medicine. Common CHM prescribed for treating angina includes Tongxinluo capsules, Fufangdanshen dripping pills, Shengmai capsules, and Shexiangbaoxin tablets. Tongxinluo capsules mainly take effect by dilating coronary arteries, increasing blood vessel perfusion flow, and strengthening cardiac contractility [10, 11]. Fufangdanshen dripping pills may alleviate angina via the antimyocardial ischemia and antiatherosclerotic effect [12, 13]. Likewise, not only can Shengmai capsules raise coronary blood flow, it may also improve the tissue tolerance to oxygen privation of cardiac muscle [14]. Apart from expanding blood vessel and increasing crown arteries current capacity, Shexiangbaoxin tablets may also alleviate arteriosclerosis and steady plague [15]. However, there is still a knowledge gap to clearly establish evidence that CHM is effective in improving the outcomes of angina patients. We therefore performed this systematic review of randomized controlled trials (RCT) to evaluate the add-on effect of CHM in angina patients.

## 2. Materials and Methods

This study was undertaken according to the recommendation of Cochrane handbook for systematic reviews of interventions [16] and reported according to the PRISMA statement [17].

### 2.1. Inclusion Criteria

Studies are eligible if (1) RCTs which compared CHM + western medicine versus western medicine, or compared CHM versus no treatment/placebo; (2) the participants were patients with stable or unstable angina (diagnosed by typical angina chest pain, and ischemic ST-segment depression by electrocardiography); (3) the intervention may be any preparations containing at least one herb that is included in the latest version of the Chinese Pharmacopeia; (4) the follow-up time should be at least 7 days. The primary outcome of this study is mortality (death from myocardial infarction (MI) and other causes). The secondary outcomes include recurrent MI, heart failure, quality of life, use of revascularization, deterioration or improvement in symptoms of angina, and adverse events. Trials were eligible if one of the hard outcomes (mortality, recurrent MI, or heart failure) was reported.

### 2.2. The Literature Search

**Figure 1:**
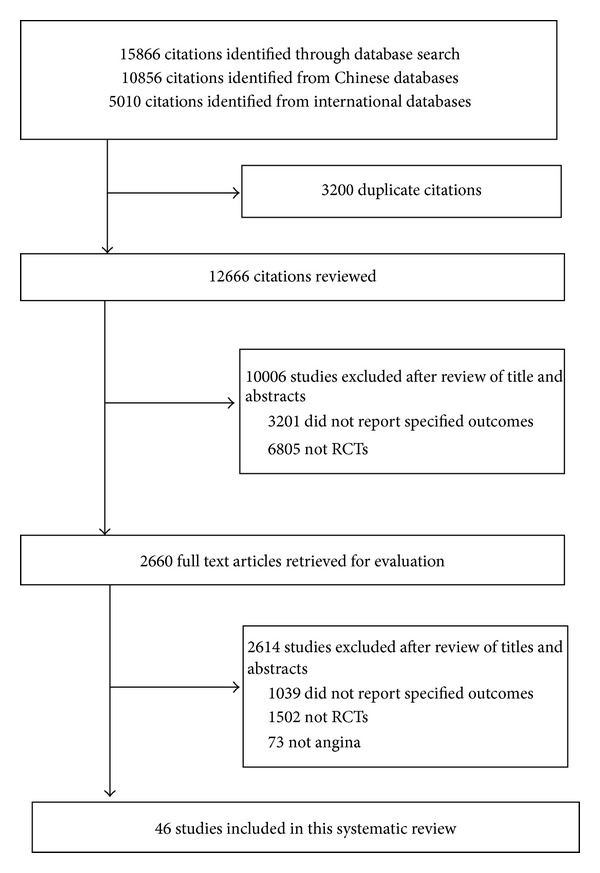
Flowchart of study selection.

We searched CENTRAL, MEDLINE, EMBASE, CINAHL, AMED, Chinese Biomedical Database (CBM), Chinese Medical Current Contents (CMCC), and Traditional Chinese Medical Literature Analysis and Retrieval System (TCMLARS) since their inception to July 2010 (Figure 1). There was no limitation on language or publication status. The search strategy included the following key words: “Chinese herbal,” “traditional Chinese medicine,” “herb,” “angina,” “stenocardia,” “clinical trials,” and “randomized controlled trial.” The reference lists of relevant trials and review articles, abstracts from major relevant conferences, and relevant trials registers were checked for additional studies.

### 2.3. Selection of Studies

Study eligibility was independently determined by two authors. All the citations were inputted into reference management software Endnote, and the duplicates were removed. The authors then evaluated the eligibility of remaining studies by examining the titles, abstracts, and full articles progressively. Discrepancies were resolved by discussion.

### 2.4. Data Extraction and Quality Assessment

Data were extracted independently by two authors using a standard form. Data extracted include (1) general information (e.g., title, authors, reference, language, year of publication, and setting); (2) trial characteristics related to methodological quality (e.g., design, duration of followup, sequence generation, allocation sequence concealment, and blinding); (3) intervention and comparison (dose, route, and timing); (4) patients (e.g., baseline characteristics and diagnostic criteria); (5) outcomes (e.g., estimates, standard error, and *P* value). Discrepancies were resolved by discussion. The authors of original studies were consulted for missing information where necessary.

The methodological quality of included randomized trials was assessed and reported by the Cochrane Collaboration's tool to assess the risk of bias [16]. The methodological quality assessed included: (1) sequence generation, (2) allocation sequence concealment, (3) blinding, (4) incomplete outcome data, (5) selective outcome reporting, and (6) other potential sources of bias.

### 2.5. Data Analysis

All analyses were conducted using the Review Manager software compiled by the Cochrane Collaboration [16]. Dichotomous outcomes were expressed as relative risk (RR). 95% confidence intervals (CIs) were calculated for all estimates. Tests for heterogeneity were performed with chi-squared test at a significance level of *P* = 0.1. *I* ^2^ statistic was calculated to estimate variation across studies. We regarded *I* ^2^ < 25% as an indicator of low heterogeneity level, 25–50% as moderate level, and >50% as high level. The estimates were pooled with a fixed-effect model if there was no significant heterogeneity. Whenever a significant heterogeneity presented, a random-effect model was used to pool the results. We intended to explore the potential sources of heterogeneity by subgroup analysis and metaregression if the number of trials was sufficient. We assessed the publication bias by funnel plot, and adjustment was made to reduce the effect of publication bias in the estimate of effectiveness.

## 3. Results

### 3.1. The Literature Search and Study Characteristics

Figure 1 showed the study selection in this study. Our search in bibliographic databases yielded 15866 citations, of which 2660 were classified as potentially relevant and were subjected to full text assessment. Finally, a total of 46 studies were included [18–63], of which 9 studies included patients with SA [18–26], 31 studies included patients with UA [27–57], and 6 studies included patients with SA or UA [58–63]. This systematic review totally included 4212 patient, with 2141 patients receiving the combination of CHM and western medicine and 2071 patients receiving western medicine alone. The duration of treatment and followup ranged from 1 day to 48 days, and 7 months to 36 months, respectively.  Table 1 demonstrated the characteristics of included studies.

### 3.2. Risk of Bias

**Figure 2:**
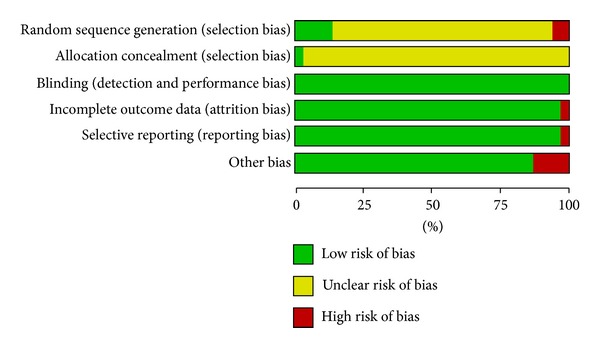
The quality of included studies.

Among included studies, only 6 trials concealed allocation sequence, two studies did not conceal the allocation sequence generated, and the rest studies were unclear. As to randomization, two studies had high risk of bias, and the rest studies had either low or uncertain risk of bias. All the included studies had low risk of bias except one study that had high risk of bias for selective outcome reporting. The details are shown in Table 2 and Figure 2.

### 3.3. Add-On Effect of Chinese Herbal Medicine in Patients with SA

**Figure 3:**
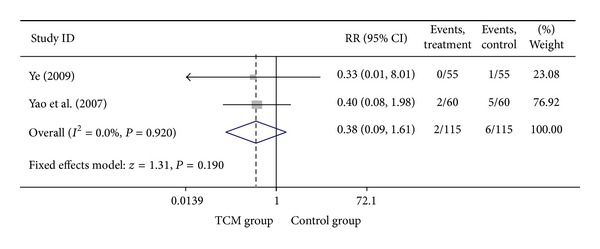
Meta-analysis of trials comparing the effect of traditional Chinese medicine with no treatment in patients with stable angina: outcome = death.

**Figure 4:**
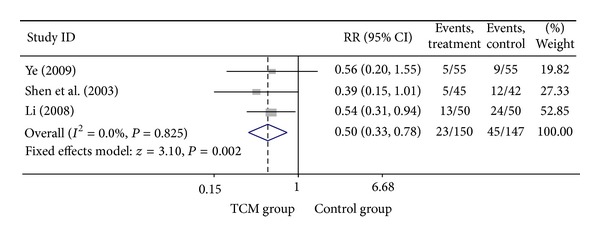
Meta-analysis of trials comparing the effect of traditional Chinese medicine with no treatment in patients with stable angina: outcome = total heart events.

**Figure 5:**
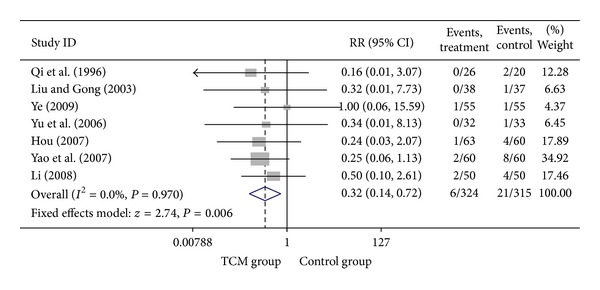
Meta-analysis of trials comparing the effect of traditional Chinese medicine with no treatment in patients with stable angina: outcome = myocardial infarction.

**Figure 6:**
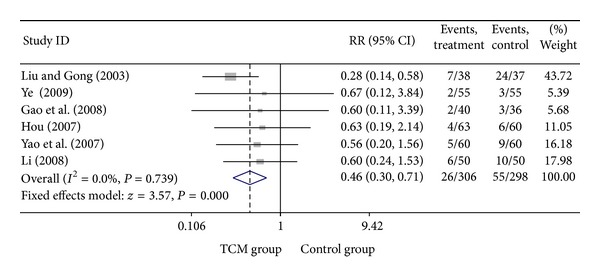
Meta-analysis of trials comparing the effect of traditional Chinese medicine with no treatment in patients with stable angina: outcome = angina.

**Figure 7:**
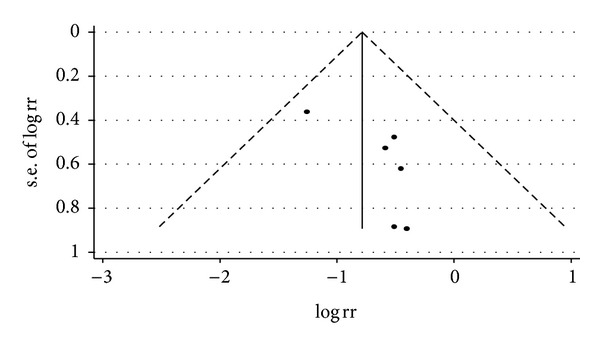
Funnel plot for relative risk of occurrence of angina between traditional Chinese medicine group and control group in patients with stable angina.

For trials that only included SA patients, we analyzed the following outcomes: total heart events (3 trials), MI (7 trials), cardiac arrhythmia (2 trials), heart failure (3 trials), angina (6 trials), and death (2 trials). All pooled results showed homogeneity (*P* > 0.1). Though the SA patients in CHM group had a lower death rate (1.74%) than SA patients in control group (5.22%), the difference was not statistically significant (*P* = 0.190). Twenty-three out of 150 SA patients who were treated with the combination of CHM with western medicine got total heart events, with an incidence rate of 15.33%, which was significantly lower (RR = 0.50, 95% CI 0.33–0.78; *P* = 0.002) than that of SA patients who were treated with western medicine alone (30.61%). Compared with western medicine alone, the combination of CHM with western medicine significantly reduced the occurrence of myocardial infarction, from 6.67% to 1.85, with the pooled RR equal to 0.32 (95% CI 0.14–0.72, *P* = 0.006). The incidences of heart failure (RR = 0.37, 95% CI 0.15–0.91; *P* = 0.031), cardiac arrhythmia (RR = 0.27, 95% CI 0.13–0.57; *P* = 0.001), and angina (RR = 0.46, 95% CI 0.30–0.71; *P* < 0.001) were also significantly lower in SA patients who were treated with the combination of CHM with western medicine than that of patients treated with western medicine alone. There were only one study that explored the difference of difference on the outcomes of fatal events, sudden cardiac death, and adverse events such as bleeding and stomach discomfort between patients in CHM group and patients in control group, none of them were different between two groups. Visual inspection of the funnel plots revealed no asymmetry except the meta-analysis of the occurrence of angina. Details of statistical results can be found in Table 3 and Figures 3, 4, 5, 6, and 7.

### 3.4. Add-On Effect of Chinese Herbal Medicine in Patients with UA

**Figure 8:**
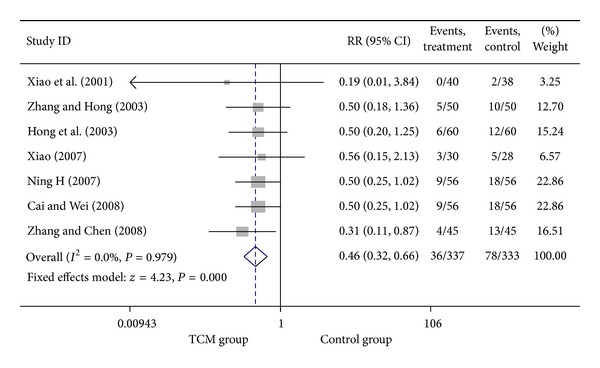
Meta-analysis of trials comparing the effect of traditional Chinese medicine with no treatment in patients with unstable angina: outcome = total heart events.

**Figure 9:**
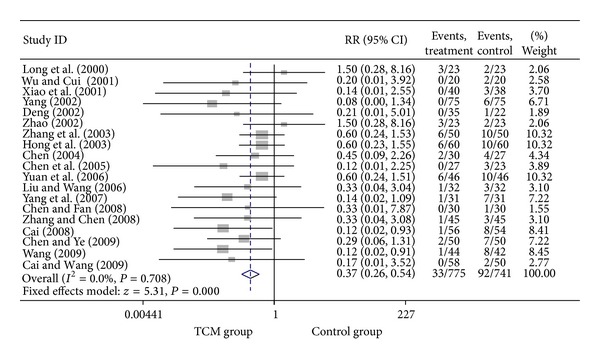
Meta-analysis of trials comparing the effect of traditional Chinese medicine with no treatment in patients with unstable angina: outcome = myocardial infarction.

**Figure 10:**
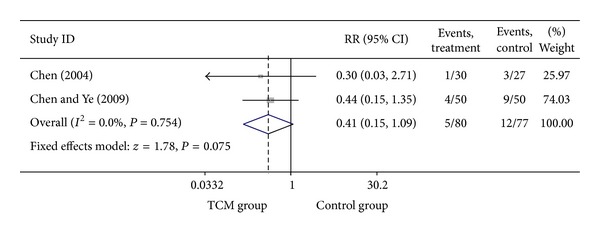
Meta-analysis of trials comparing the effect of traditional Chinese medicine with no treatment in patients with unstable angina: outcome = heart failure.

**Figure 11:**
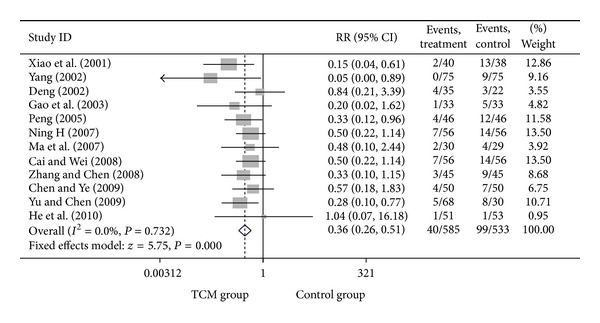
Meta-analysis of trials comparing the effect of traditional Chinese medicine with no treatment in patients with unstable angina: outcome = angina.

**Figure 12:**
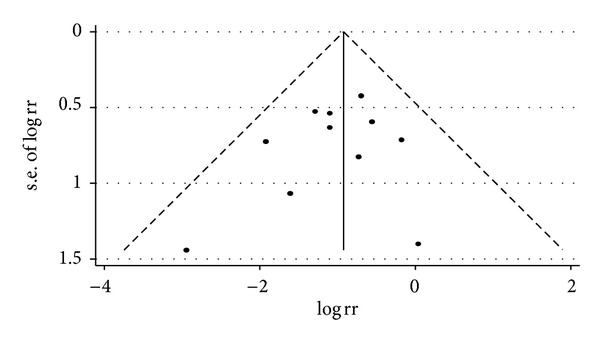
Funnel plot for relative risk of occurrence of angina between traditional Chinese medicine group and control group in patients with unstable angina.

For trials that only included UA patients, we analyzed the following outcomes: total heart events (7 trials), death (1 trial), MI (19 trials), cardiac arrhythmia (1 trial), heart failure (2 trials), angina (12 trials), need for cardiac surgery (2 trials), and need for PCI (3 trials). All pooled results showed homogeneity. There are 337 patients in CHM group, of whom 36 patients developed total heart events (10.68%), while 78 out of 333 patients (23.42%) in the control group occurred total heart events. Patients in CHM group had a significant lower incidence of total heart events than patients in the control group, with a pooled RR equal to 0.46 (95% CI 0.32–0.66, *P* < 0.001). Compared with western medicine alone, the combination of CHM with western medicine significantly reduced the occurrence of myocardial infarction, from 12.42% in control group to 4.26% in CHM group, with a pooled RR of 0.37 (95% CI 0.26–0.54, *P* < 0.001). Patients in the CHM group had significant lower heart events (RR = 0.46, 95% CI 0.32–0.66; *P* < 0.001), myocardial infarction (RR = 0.37, 95% CI 0.26–0.54; *P* < 0.001), angina (RR = 0.36, 95% CI 0.26–0.51; *P* < 0.001), and sudden cardiac death (RR = 0.22, 95% CI 0.06–0.84; *P* = 0.027) than patients in control group. Pooled result showed no significant difference on death (RR = 0.19, 95% CI 0.01–3.84; *P* = 0.279), heart failures (RR = 0.41, 95% CI 0.15–1.09; *P* = 0.075) and fatal events (RR = 0.33, 95% CI 0.07–1.62; *P* = 0.173) between groups. Two studies presented the data on the adverse events on the treatment, and there is no significant difference on bleeding and stomach discomfort between groups. Visual inspection of funnel plots suggesting there's no publication bias. Details of statistical results can be found in Table 3 and Figures 8, 9, 10, 11 and 12.

### 3.5. Add-On Effect of Chinese Herbal Medicine in Patients with SA and UA

**Figure 13:**
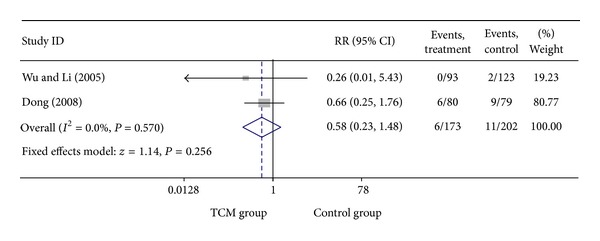
Meta-analysis of trials comparing the effect of traditional Chinese medicine with no treatment in patients with angina: outcome = death.

**Figure 14:**
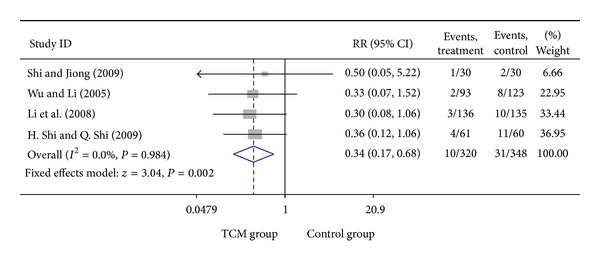
Meta-analysis of trials comparing the effect of traditional Chinese medicine with no treatment in patients with angina: outcome = myocardial infarction.

**Figure 15:**
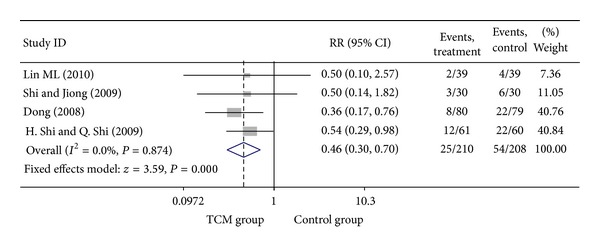
Meta-analysis of trials comparing the effect of traditional Chinese medicine with no treatment in patients with angina: outcome = angina.

**Figure 16:**
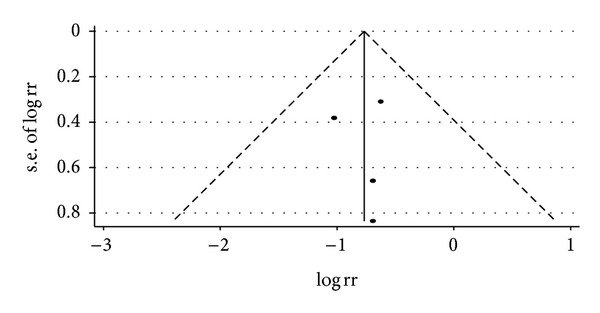
Funnel plot for relative risk of occurrence of angina between traditional Chinese medicine group and control group in patients with angina.

For trials including patients with diagnosis of either SA or UA, we analyzed the following outcomes: total heart events (1 trial), MI (4 trials), cardiac arrhythmia (1 trial), heart failure (2 trials), angina (4 trials), need for cardiac surgery (1 trial), and death (2 trials). All pooled results showed homogeneity. Total death rate in CHM group was 3.47% (6/173), compared with 5.44% (11/202) for the control group. The result showed that patients treated with CHM did not have a significant lower death rate than no-CHM treatment patients group, with a pooled RR of 0.58 (95% CI 0.23–1.48; *P* = 0.256). There was no evidence of statistical heterogeneity across the studies using the *I* ^2^ test (*I* ^2^ = 0, *P* = 0.570). Meta-analysis of trials comparing the effect of CHM with no CHM treatment in patients with either SA or UA showed that the myocardial infarction rate was 3.13% (10/320) and 8.91% (31/348) in CHM treatment group and control group, respectively, with a pooled RR of 0.34 (95% CI 0.17–0.68; *P* = 0.002). There was enough evidence to illustrate that patients with CHM treatment had a significant lower myocardial infarction rate than the control group. No evidence of statistical heterogeneity across the studies was identified using the *I* ^2^ test (*I* ^2^ = 0, *P* = 0.984 > 0.1). Meta-analysis showed that patients with CHM treatment had a significant lower angina rate than no CHM treatment group, with a pooled RR of 0.46 (95% CI 0.30–0.70; *P* = 0.000). There was no evidence of statistical heterogeneity (*I* ^2^ = 0, *P* = 0.874 > 0.1). Visual inspection of funnel plots suggesting no publication bias. Details of statistical results can be found in Table 3 and Figures 13, 14, 15 and 16.

## 4. Discussion

This systematic review synthesized evidence from 4212 patients in 46 RCTs. The main findings of this systematic review are as follows. (1) The combination of CHM with western medicine may significantly reduce the occurrence of total heart events, MI, cardiac arrhythmia, heart failure, and angina compared with western medicine alone in patients with SA. (2) For patients with UA, the combination therapy is superior to western medicine alone on total heart events, myocardial infarction, angina, and sudden cardiac death. But there is no significant difference on the incidence of death, cardiac arrhythmia, hart failure, fatal events, cardiac surgery, and percutaneous coronary intervention. (3) For patients with SA or UA, the combination therapy may lead to significant lower occurrence of myocardial infarction, heart failure, and angina as compared with western medicine monotherapy, but no significant differences were indentified on the incidence of total heart events, death, cardiac arrhythmia, fatal events, sudden cardiac death, cardiac operation, bleeding, and stomach discomfort.

Apart from this study, the add-on benefits of CHM on top in western medicine treatment in angina patients were also studied in some other systematic reviews [64]. It was suggested that Danshen preparation [65, 66], Dengzhanhua injection [67], Tongxinluo capsule [68, 69], Shuxuetong [70], Ginkgo extract [71, 72], and Compound salvia pellet [73, 74] would be beneficial for angina patients. These systematic reviews usually only focused on one type of CHM [15, 65–67, 69–73], included only a limited number of original studies [65, 67, 70], and did not receive update for a long time [66, 67, 69], and their conclusions may therefore be influenced.

The overall risk of bias of included studies was moderate. Inadequacy in reporting methods for randomization and allocation concealment is a major problem among most included RCTs, which may lead to selection bias in our study [16]. The lack of blinding is another problem in most included trials; however, the impact on conclusion was less critical as we focused on objective outcomes. Visual inspection of funnel plots indicated no symmetry on most outcomes, except for the meta-analysis of angina in patients with SA. We therefore believe publication bias is unlikely to be a serious threat to the estimates.

Apart from the limitations on the quality of included studies, the estimates of some outcomes, such as the incidence of fatal events, sudden cardiac death, and bleeding in patients with UA are also limited by relatively small sample size. For these outcomes, only one study with less than 100 patients was included in meta-analysis, and the precision of estimates was therefore influenced [75].

To the best of our knowledge, this is the most comprehensive assessment of add-on effect of CHM in patients with angina. We employed a contemporaneous search strategy in both international and Chinese databases to ensure most studies were included. This enabled us to locate a much higher number of studies compared to other existing reviews on this topic. Additionally, the study selection, data extraction, and quality assessment in this study were independently carried out by two authors to ensure high validity.

This study has important implication for clinical practice and CHM research. For practitioners, this systematic review demonstrated consistent, add-on benefits of using CHM on top in western medicine treatment for preventing all-cause and cardiac mortality amongst angina patients. However, this conclusion was drawn from moderate or low quality trials and the estimates on some outcomes were based on limited number of patients. Large scale, rigorously designed RCTs are still wanted to confirm these conclusions.

## Figures and Tables

**Table 1:** Main characteristic of included studies.

Study	Diagnosis	Patient no. intervention/control	Treatment		Treatment duration (days)	Followup (months)
			Intervention	Control		
Qi et al [18], 1996	SA	26/20	Shexiangbaoxin tablets + WM	WM	14	NR
Liu and Gong [19], 2003	SA	38/37	Shengmai capsules + WM	WM	NR	6
Shen et al. [20], 2003	SA	45/42	Fufangdanshen dripping pills +	WM	NR	36
Yu et al. [21], 2006	SA	32/33	Xuezhikang capsules + WM	WM	180	6
Hou [22], 2007	SA	63/60	Danhong In + WM	WM	14	1
Yao et al. [23], 2007	SA	60/60	Weiaoxin + WM + placebo	WM + placebo	30	12
Gao et al. [24], 2008	SA	40/36	Yiqihuoxuefang + WM without antiplatelet drug	WM without antiplatelet drug	30	1
Li [25], 2008	SA	50/50	Dengzhanshengmai capsules + WM	WM	360	12
Ye [26], 2009	SA	55/55	Xinyuan capsules + Simvastahn	Simvastahn	365	12
Long et al. [27], 2000	UA	23/23	Tongxinluo capsules + WM	WM	NR	NR
Wu and Cui [28], 2001	UA	20/20	Shuxuetong In + WM	WM	10	NR
Xiao et al. [29], 2001	UA	40/38	Xuezhikang capsules + WM	WM	90	3
Deng [30], 2002	UA	35/22	Ziniguanxintong decoction + WM	WM	58	2
Yang [31], 2002	UA	75/75	Ruxinan capsules + WM	WM	21	3
Zhao [32], 2002	UA	23/23	Xuesaitong pills + WM	WM	NR	NR
Gao et al [33], 2003	UA	33/33	Xintong oral liquid + WM	WM	90	3
Hong et al [34], 2003	UA	60/60	Fufangdanshen dripping pills + WM	WM	28	6
Nian [35], 2003	UA	15/15	Tongxinluo capsules + WM	WM	28	1
Zhang and Hong [36], 2003	UA	50/50	Fufangdanshen dripping pills + WM	WM	28	6
Chen [37], 2004	UA	30/27	Shexiangbaoxin pills + WM	WM	28	48
Chen et al. [38], 2005	UA	27/23	Ciwujia In + WM	WM	14	3
Peng [39], 2005	UA	46/46	Shuxuening In + WM + aspirin + dinitrosorbide	WM + aspirin + dinitrosorbide	NR	NR
Liu and Wang [40], 2006	UA	32/32	Tongxinluo capsules + WM	WM	56	2
Yuan et al. [41], 2006	UA	46/46	Shuxuening In + WM	WM	14	6
Ning et al. [42], 2007	UA	56/56	Tanshinone II A sulfoacid In + WM	WM	14	6
Ma et al. [43], 2007	UA	30/29	Tanshinone II A sulfoacid In + WM + dinitrosorbide	WM + dinitrosorbide	14	1
Xiao [44], 2007	UA	30/28	Chaihuxianxiong decoction + WM	WM	30	6
Yang et al. [45], 2007	UA	31/31	Buxuzhuyuheji + WM	WM	14	6
Cai and Wei [46], 2008	UA	56/56	Danshendongganfen + WM	WM	14	6
Cai [47], 2008	UA	56/54	Gegensu capsules + WM	WM	14	2
Chen and Fan [48], 2008	UA	30/30	Xintongfang + WM + dinitrosorbide	WM + dinitrosorbide	28	1
Liu [49], 2008	UA	31/31	Gegensu In + WM	WM	14	3
Zhang and Chen [50], 2008	UA	45/45	Tongxinluo capsules + WM	WM	NR	1.5
Cai and Wang [51], 2009	UA	58/50	Tongxinluo capsules + WM + dinitrosorbide	WM + dinitrosorbide	28	1
Chen and Ye [52], 2009	UA	50/50	Dengzhanshengmai capsules+ WM	WM	180	6
Su et al. [53], 2009	UA	60/58	Danhong In + WM	WM	14	NR
Wang [54], 2009	UA	44/42	Gegensu In + WM	WM	14	2
Wang et al. [55], 2009	UA	37/34	Shuxuetong In + WM	WM	7	NR
Yu and Chen [56], 2009	UA	68/30	Tongxinluo capsules + WM	WM	NR	3
He et al. [57], 2010	UA	54/54	Yiqiyangyinfang + WM	WM	28	8
Wu and Li [58], 2005	SA or UA	93/123	Tongxinluo capsules + WM	WM	14	0.5
Li et al. [59], 2008	SA or UA	136/135	Quyuningxin WM pills + WM	WM	14	0.5
Dong [60], 2008	SA or UA	80/79	Shexiangbaoxin pills + WM + dinitrosorbide	WM + dinitrosorbide	90	3
H. Shi and Q. Shi [61], 2009	SA or UA	63/61	Fufangdanshen dripping pills + WM	WM	NR	12
Shi and Jiang [62], 2009	SA or UA	30/30	Tongxinluo capsules + WM atorvastatin	WM atorvastatin	180	6
Lin and Wu [63], 2010	SA or UA	39/39	Fufangdanshen dripping pills + WM	WM	28	6

SA: stable angina; UA: unstable angina; WM: west medicine; NR: not reported.

**Table 2:** Methodological qualities of the included studies.

Study	Risk of bias for randomization	Risk of bias for concealment	Risk of bias for blinding	Risk of bias for incomplete data	Risk of bias for selective outcome reporting	Risk bias for other problems
Qi et al [18], 1996	Uncertain	Uncertain	Low risk	Low risk	Low risk	Low risk
Liu and Gong [19], 2003	Uncertain	Uncertain	Low risk	Low risk	Low risk	Low risk
Shen et al. [20], 2003	High risk	Uncertain	Low risk	Low risk	Low risk	Low risk
Yu et al. [21], 2006	Low risk	Uncertain	Low risk	Low risk	Low risk	Low risk
Hou [22], 2007	Uncertain	Uncertain	Low risk	Low risk	Low risk	Low risk
Yao et al. [23], 2007	Uncertain	Uncertain	Low risk	Low risk	Low risk	Low risk
Gao et al. [24], 2008	Low risk	Uncertain	Low risk	Low risk	Low risk	Low risk
Li [25], 2008	Uncertain	Uncertain	Low risk	Low risk	High risk	Low risk
Ye [26], 2009	Uncertain	Uncertain	Low risk	Low risk	Low risk	Low risk
Long et al. [27], 2000	Uncertain	Uncertain	Low risk	Low risk	Low risk	Low risk
Wu and Cui [28], 2001	Uncertain	Uncertain	Low risk	Low risk	Low risk	Low risk
Xiao et al. [29], 2001	High risk	Uncertain	Low risk	Low risk	Low risk	High risk
Deng [30], 2002	Uncertain	Uncertain	Low risk	Low risk	Low risk	Low risk
Yang [31], 2002	Uncertain	Uncertain	Low risk	Low risk	Low risk	Low risk
Zhao [32], 2002	Uncertain	Uncertain	Low risk	Low risk	Low risk	High risk
Gao et al [33], 2003	Uncertain	Uncertain	Low risk	Low risk	Low risk	Low risk
Hong et al [34], 2003	Uncertain	Uncertain	Low risk	Low risk	Low risk	High risk
Nian [35], 2003	Uncertain	Uncertain	Low risk	Low risk	Low risk	Low risk
Zhang and Hong [36], 2003	Uncertain	Uncertain	Low risk	Low risk	Low risk	High risk
Chen [37], 2004	Uncertain	Uncertain	Low risk	Low risk	Low risk	Low risk
Chen et al. [38], 2005	Uncertain	Uncertain	Low risk	Low risk	Low risk	Low risk
Peng [39], 2005	Uncertain	Uncertain	Low risk	Low risk	Low risk	Low risk
Liu and Wang [40], 2006	Uncertain	Uncertain	Low risk	Low risk	Low risk	Low risk
Yuan et al. [41], 2006	Uncertain	Uncertain	Low risk	Low risk	Low risk	Low risk
Ning et al. [42], 2007	Low risk	Uncertain	Low risk	Low risk	Low risk	Low risk
Ma et al. [43], 2007	Uncertain	Uncertain	Low risk	Low risk	Low risk	Low risk
Xiao [44], 2007	Low risk	Uncertain	Low risk	Low risk	Low risk	Low risk
Yang et al. [45], 2007	Uncertain	Uncertain	Low risk	Low risk	Low risk	Low risk
Cai and Wei [46], 2008	Low risk	Uncertain	Low risk	Low risk	Low risk	Low risk
Cai [47], 2008	Uncertain	Uncertain	Low risk	Low risk	Low risk	Low risk
Chen and Fan [48], 2008	Uncertain	Uncertain	Low risk	Low risk	Low risk	Low risk
Liu [49], 2008	Uncertain	Uncertain	Low risk	Low risk	Low risk	Low risk
Zhang and Chen [50], 2008	Uncertain	Uncertain	Low risk	Low risk	Low risk	Low risk
Cai and Wang [51], 2009	Uncertain	Uncertain	Low risk	Low risk	Low risk	Low risk
Chen and Ye [52], 2009	Uncertain	Uncertain	Low risk	High risk	Low risk	Low risk
Su et al. [53], 2009	Uncertain	Uncertain	Low risk	Low risk	Low risk	Low risk
Wang [54], 2009	Uncertain	Uncertain	Low risk	Low risk	Low risk	High risk
Wang et al. [55], 2009	Uncertain	Uncertain	Low risk	Low risk	Low risk	Low risk
Yu and Chen [56], 2009	Uncertain	Uncertain	Low risk	Low risk	Low risk	Low risk
He et al. [57], 2010	Low risk	Low risk	Low risk	Low risk	Low risk	Low risk
Wu and Li [58], 2005	Uncertain	Uncertain	Low risk	Low risk	Low risk	Low risk
Li et al. [59], 2008	Uncertain	Uncertain	Low risk	Low risk	Low risk	Low risk
Dong [60], 2008	Uncertain	Uncertain	Low risk	Low risk	Low risk	Low risk
H. Shi and Q. Shi [61], 2009	Uncertain	Uncertain	Low risk	Low risk	Low risk	Low risk
Shi and Jiang [62], 2009	Uncertain	Uncertain	Low risk	Low risk	Low risk	Low risk
Lin and Wu [63], 2010	Uncertain	Uncertain	Low risk	Low risk	Low risk	High risk

**Table 3:** Chinese herbal medicine plus western medicine versus western medicine alone for treating stable angina: meta-analysis results.

Events	No. of studies	No. of events/total no.		Combined effect		Heterogeneity		
		CHM	Control	RR (95% CI)	*P* value	*Q* value	*P* value	*I* ^2^
Stable angina								
Total heart events	3	23/150	45/147	0.50 (0.33–0.78)	0.002	0.39	0.825	0.0
Death	2	2/115	6/115	0.38 (0.09–1.61)	0.190	0.01	0.920	0.0
Myocardial infarction	7	6/324	21/315	0.32 (0.14–0.72)	0.006	1.34	0.970	0.0
Cardiac arrhythmia	2	7/93	25/92	0.27 (0.13–0.57)	0.001	0.28	0.594	0.0
Heart failure	3	6/168	16/165	0.37 (0.15–0.91)	0.031	0.27	0.875	0.0
Angina	6	26/306	55/298	0.46 (0.30–0.71)	<0.001	2.75	0.739	0.0
Fatal events	1	1/45	2/42	0.47 (0.04–4.96)	0.527	—	—	—
Sudden cardiac death	1	2/50	3/50	0.67 (0.12–3.82)	0.649	—	—	—
Bleeding	1	1/40	1/36	0.90 (0.06–13.87)	0.940	—	—	—
Stomach discomfort	1	2/55	4/55	0.50 (0.10–2.62)	0.653	—	—	—
Unstable angina								
Total heart events	7	36/337	78/333	0.46 (0.32–0.66)	<0.001	1.15	0.979	0.0
Death	1	0/40	2/38	0.19 (0.01–3.84)	0.279	—	—	—
Myocardial infarction	19	33/775	92/741	0.37 (0.26–0.54)	<0.001	14.32	0.708	0.0
Cardiac arrhythmia	1	5/50	10/50	0.50 (0.18–1.36)	0.174	—	—	—
Heart failure	2	5/80	12/77	0.41 (0.15–1.09)	0.075	0.10	0.754	0.0
Angina	12	40/585	99/533	0.36 (0.26–0.51)	<0.001	7.79	0.732	0.0
Fatal events	4	0/147	4/147	0.33 (0.07–1.62)	0.173	0.00	1.000	0.0
Sudden cardiac death	5	0/243	9/243	0.22 (0.06–0.84)	0.027	0.08	0.999	0.0
Cardiac surgery	2	2/56	7/56	0.29 (0.06–1.32)	0.108	0.33	0.564	0.0
PCI	3	3/118	9/117	0.35 (0.11–1.16)	0.088	0.96	0.618	0.0
Bleeding	1	1/31	11/31	0.09 (0.01–0.66)	0.018	—	—	—
Stomach discomfort	2	1/63	3/63	0.67 (0.11–3.90)	0.653	1.49	0.222	32.8
Stable angina or unstable angina								
Total heart events	1	3/39	7/39	0.43 (0.12–1.54)	0.194	—	—	—
Death	2	6/173	11/202	0.58 (0.23–1.48)	0.256	0.32	0.570	0.0
Myocardial infarction	4	10/320	31/348	0.34 (0.17–0.68)	0.002	0.46	0.984	0.0
Cardiac arrhythmia	1	2/30	4/30	0.50 (0.10–2.53)	0.402	—	—	—
Heart failure	2	12/110	26/109	0.46 (0.24–0.86)	0.015	0.21	0.646	0.0
Angina	4	25/210	54/208	0.46 (0.30–0.70)	<0.001	0.70	0.874	0.0
Fatal events	2	4/110	9/109	0.44 (0.14–1.39)	0.161	0.01	0.903	0.0
Sudden cardiac death	1	1/61	3/60	0.33 (0.04–3.06)	0.328	—	—	—
Cardiac operation	1	8/80	16/79	0.49 (0.22–1.09)	0.080	—	—	—
Bleeding	1	1/40	1/36	0.90 (0.06–13.87)	0.940	—	—	—
Stomach discomfort	3	8/130	2/129	2.99 (0.83–10.73)	0.094	1.04	0.594	0.0

CHM: chinese herbal medicine; RR: risk ratio; CI: confidence interval; PCI: percutaneous transluminal coronary intervention.
